# Medical complexity and time to lung cancer treatment – a three-year retrospective chart review

**DOI:** 10.1186/s12913-016-1952-y

**Published:** 2017-01-17

**Authors:** Trine Stokstad, Sveinung Sørhaug, Tore Amundsen, Bjørn H. Grønberg

**Affiliations:** 1 Department of Cancer Research and Molecular Medicine, NTNU, Norwegian University of Science and Technology, PO Box 8905, N-7491 Trondheim, Norway; 2 Department of Gynecology, St. Olavs Hospital – Trondheim University Hospital, Trondheim, Norway; 3 Department of Circulation and Medical Imaging, NTNU, Norwegian University of Science and Technology, Trondheim, Norway; 4 Department of Thoracic Medicine, St. Olavs Hospital – Trondheim University Hospital, Trondheim, Norway; 5 Cancer Clinic, St. Olavs Hospital – Trondheim University Hospital, Trondheim, Norway

**Keywords:** Quality indicator, Organization, Performance, Timeliness, Complexity

## Abstract

**Background:**

The time from a referral for suspected lung cancer is received at a hospital until treatment start has been defined as a quality indicator. Current Norwegian recommendation is that ≥70% should start surgery or radiotherapy within 42 calendar days and systemic therapy within 35 days. However, delays can occur due to medical complexity. The aim of this study was to quantify the proportion of patients who started treatment within the recommended timeframes; and to assess the proportion of non-complex patients for which there were no good reasons for delays.

**Methods:**

We performed a retrospective chart review of all patients diagnosed with lung cancer at a university hospital during 2011–2013. We defined “non-complex” patients as those who underwent ≤1 tissue diagnostic procedure and had no delays due to comorbidity, intercurrent disease or complications to diagnostic procedures (“Medical delays”) of more than three days.

**Results:**

Four hundred forty-nine cases were analyzed; 142 (32%) had >1 tissue diagnostic procedures; 67 (15%) had medical delays >3 days; 262 (58%) were non-complex and 363 (81%) received treatment for lung cancer. Median number of days until surgery or radiotherapy was 48 (overall) and 41 (non-complex patients). The proportions who started surgery or radiotherapy within 42 days were 41% (overall) and 56% (non-complex). Corresponding numbers for systemic therapy were 29 days (overall) and 25 days (non-complex), and 64% (overall) and 80% (non-complex).

**Conclusion:**

Fewer lung cancer patients than desired started treatment within the recommended timeframes. Even among the least complex patients, too few patients received timely treatment. The reasons need to be identified and understood, and changes in the organization appear to be necessary in order to offer timely treatment to more patients.

**Electronic supplementary material:**

The online version of this article (doi:10.1186/s12913-016-1952-y) contains supplementary material, which is available to authorized users.

## Background

Waiting while undergoing investigations for suspected cancer is distressing for patients and their families [1–3], and waiting for cancer treatment to start is perceived as a medical risk that may affect treatment outcomes [4, 5]. It is not clear that shorter time to treatment influences survival [6–9], but there is fair evidence that prompt management improves patient satisfaction and reduces anxiety [1, 2, 10–12]. Thus, efficient organization of cancer diagnosis and treatment is a public and political goal. Political strategies to improve organization include development of indicators and standards for timely diagnosis and treatment.

The British Thoracic Society and the Danish Lung Cancer Group presented the first specifications for timely lung cancer diagnosis and treatment in 1998 [8, 13]. In June 2011, the first Norwegian recommendations regarding timelines for diagnosis and treatment of cancer were presented. At that time, at least 80% of all cancer patients were to start treatment within 20 working days from a referral letter for suspected cancer was received at a hospital. National guidelines for cancer care organization were developed, and the National Standard for lung cancer diagnosis and treatment was presented on January 1, 2015 [14]. For suspected lung cancer, the first hospital appointment should be offered within seven calendar days of receiving a referral letter; a treatment decision should be made within 28 calendar days; systemic therapy should start within 35 calendar days, and surgery or radiotherapy within 42 calendar days. The overall aim is that more than 70% of lung cancer patients start treatment within these timeframes [15].

The metrics for timely lung cancer care vary between health care organizations [16–18], but all accept that good reasons for delay exist and tolerate longer timeframes for a specified proportion of patients. Diagnostic workup for lung cancer may be complex and it is not clear whether it is realistic or medically correct that all patients start treatment within 35 or 42 days. Tissue sampling may be difficult; the number of lesions that should be punctured varies; and complications to diagnostic procedures occur. Many patients are elderly and suffer from co-existing conditions. Thus, intercurrent diseases are common, and some patients want breaks between the diagnostic procedures or before starting treatment [19]. Most studies do not consider these factors, since they are based on registry data. Thus, there is no established method for assessing complexity in diagnostic work-up for lung cancer [20].

### Aims of the study

The main aims of the study were to investigate how many patients at a university hospital who started treatment for lung cancer within the timeframes recommended in Norway; and to quantify the proportion of patients who had delays due to complex diagnostic workup, intercurrent disease or patients’ wish.

## Methods

### Study setting

The Norwegian health care is mainly public, and the national health insurance system cover expenses exceeding € 233 per year [21]. Approximately 700 000 people live in Central Norway. There are seven hospitals in the region. All hospitals diagnose lung cancer and offer systemic therapy. Radiotherapy is offered at two sites. Complex cases are referred to St. Olavs Hospital, which is the university hospital in the region, but also serves as the primary hospital for 380 000 inhabitants. Most patients within the primary catch-up area lives within 30 min from the hospital. St. Olavs Hospital has all facilities for diagnostic workup for lung cancer including the only PET CT (Positron Emission Tomography Computer Tomography) scanner in the region (since October 2013), and all lung cancer surgery is performed here. PET CT was performed outside our health region during most of the study period (until October 2013). From 2009 to 2013, the annual world standardized lung- and tracheal cancer rate in Norway was 34.9 in men and 26.0 in women [22]. The annual incidence in the primary catchment area of St. Olavs Hospital was similar to the incidence in all of Norway.

The Department of Thoracic Medicine is responsible of lung cancer diagnosis and they offer systemic therapy. The Cancer Department provide radiotherapy, and surgery takes place in the Department of Cardio-Thoracic Surgery. Diagnostic workup for lung cancer is mainly done on an outpatient basis. A weekly, regional, multidisciplinary tumor board meeting is held between pulmonary physicians, thoracic surgeons, an oncologist specializing in lung cancer (Norwegian oncologists are trained in both medical oncology and radiotherapy), a thoracic radiologist, a specialist in nuclear medicine, a pathologist and a nurse coordinator. Between September 1, 2012 and January 31, 2013, the multidisciplinary team revised the routines and procedures for lung cancer diagnosis and a standardized care pathway was developed that included the national recommendations for timeliness. The pathway did not include protocols that could limit timeliness. They also assigned a pulmonary phycician specializing in diagnosis, staging and treatment of lung cancer as the leader of the multidisciplinary team.

### Study design

The study is a retrospective analysis of all cases that started diagnostic work-up and were diagnosed with lung cancer from January 1, 2011 to December 31, 2013, at the Department of Thoracic Medicine at St. Olavs Hospital – Trondheim University Hospital, Trondheim, Norway.

### Case selection and data collection

Patients registered with ICD 10 codes C34.0-9 (“lung cancer”) were identified from the hospital patient administrative system. Patient data were collected from the hospital electronic medical records.

Stage of disease was assessed according to the 7th edition of the TNM classification of lung cancer [23]. Patients were classified as having non-small-cell lung cancer (NSCLC); small-cell lung cancer (SCLC); other primary lung cancers; or no tissue diagnosis. Treatment was classified as curative treatment (surgery, radical radiotherapy or radio-chemotherapy of stage I-III disease); palliative treatment; or no cancer treatment/death before start of treatment. First treatment was either surgery or radiotherapy, or systemic therapy (including when chemotherapy was administered concurrently with radiotherapy). Patients were classified as “hospitalized” when admitted to the hospital due to the patient’s condition at start of diagnostic work-up, otherwise they were classified as “outpatient”.

### Complexity

In our experience, the factors that influence the timelines the most are the number of tissue diagnostic procedures required [24–27], and delays for medical reasons [27, 28].

Tissue diagnostic procedures are performed to diagnose lung cancer; to do molecular and histopathological classification; and to assess extent of disease. These procedures include bronchoscopy; endobronchial ultrasound-guided trans-bronchial needle aspiration (EBUS TBNA); trans-thoracic needle biopsy; or others. Delays in diagnostic workup for medical reasons were categorized as hospitalization caused by complications to a diagnostic procedure; synchronous investigation for other cancer; synchronous treatment of other cancer; treatment of comorbidity; or intercurrent disease.

We defined “non-complex patients” as having undergone ≤1 tissue diagnostic procedure and having no medical delays of >3 days. “complex patients” were subclassified as having >1 tissue diagnostic procedures and no medical delays of >3 days; ≤1 tissue diagnostic procedure and medical delays of >3 days; or >1 tissue diagnostic procedures and medical delays of >3 days.

### Intervals

We defined start time as the date when a referral letter for suspected lung cancer was received by the Department of Thoracic Medicine – or the date when the decision was made to start diagnostic workup in patients with a known single pulmonary nodule (SPN). We defined the time for treatment decision as the date when such a decision was documented in the EMR. We defined start of treatment as date of surgery, first fraction of radiotherapy, first day of intra-venous chemotherapy, or date of prescription of oral cancer therapy. Time to treatment treatment was defined as the number of calendar days from start time until start of treatment.

According to Norwegian recommendations, start of treatment within 42 days (surgery or radiotherapy) or 35 days (systemic therapy) was considered “timely treatment” [14].

### Statistical analyses

We used chi-square test for univariate analysis. Factors influencing the likelihood of timely treatment (patient and disease characteristics as well as PET CT – since PET CT was not available at St. Olavs Hospital during most of the study period) were explored using logistic regression analysis. We used the Stata/IC 13.1 package for Windows for the statistical analyses, and considered a p-value of < 0.05 to be statistically significant.

## Results

### Case selection and baseline characteristics

Nine hundred ninety patients were identified with “lung cancer” for the first time in the hospital registry in the study period. Four hundred three started diagnostic workup in other hospitals (*n* = 333) or other departments at St. Olavs Hospital (*n* = 34); 66 were diagnosed before January 1, 2011; 103 patients did not have lung cancer, and five patients declined to participate in the study. Thus, 449 patients were analyzed. St. Olavs Hospital was the primary hospital for 436 (97%) of these.

The proportion at an age ≥70 was higher in 2013 (67%) than in 2011 (52%) and 2012 (57%) (*p* = 0.04) due to a variation in the proportions <70 and 70–74. The proportion aged 75 or higher was stable. The proportion who underwent PET CT increased from 10% in 2011, 36% in 2012 to 51% in 2013 (*p* < 0.0001). Otherwise, there were no significant variations in baseline characteristics or treatment between 2011, 2012 and 2013. 42% received curative treatment, 39% palliative, and 18% received no cancer treatment. Seven patients (1.6%) died before a treatment started (Table 1).

**Table 1:** Baseline characteristics

Variables	Total	2011	2012	2013	Non-complex patients^a^	Complex patients^b^
	*N* = 469	*n* = 147	*n* = 146	*n* = 156	*n* = 262	*n* = 187
Age, median (range)	72 (40–93)	70 (40–90)	71 (46–91)	73 (54–93)	72 (46–93)	72 (40–89)
Age ≥ 70 years, *n* (%)	265 (59%)	77 (52%)	84 (57%)	104 (67%)	155 (59%)	110 (59%)
Women	206 (46%)	62 (42%)	76 (52%)	68 (44%)	125 (48%)	81 (43%)
TNM stage						
I	112 (25%)	29 (20%)	39 (27%)	44 (28%)	65 (25%)	47 (25%)
II	42 (9%)	19 (13%)	10 (7%)	13 (8%)	18 (7%)	24 (13%)
III	116 (26%)	43 (29%)	34 (23%)	39 (25%)	68 (26%)	48 (26%)
IV	179 (40%)	56 (38%)	63 (43%)	60 (38%)	111 (42%)	68 (36%)
Histology						
NSCLC	312 (69%)	105 (71%)	110 (75%)	97 (62%)	161 (61%)	151 (81%)
SCLC	65 (14%)	18 (12%)	19 (13%)	28 (18%)	49 (19%)	16 (9%)
Other primary lung cancers	9 (2%)	2 (1%)	1 (1%)	6 (4%)	4 (2%)	5 (3%)
No tissue diagnosis	63 (14%)	22 (15%)	17 (11%)	25 (16%)	48 (18%)	15 (8%)
Treatment						
Surgery	116 (26%)	37 (25%)	39 (27%)	40 (26%)	59 (23%)	57 (30%)
Curative radiotherapy^c^	74 (16%)	18 (12%)	22 (15%)	34 (22%)	46 (18%)	28 (15%)
Palliative radiotherapy	48 (11%)	19 (13%)	15 (10%)	14 (9%)	26 (10%)	22 (12%)
Palliative systemic therapy	120 (27%)	38 (26%)	43 (29%)	39 (25%)	69 (26%)	51 (27%)
Palliative surgery	5 (1%)	1 (1%)	1 (1%)	3 (2%)	2 (1%)	3 (2%)
No cancer treatment	79 (18%)	31 (21%)	24 (16%)	24 (15%)	55 (21%)	24 (13%)
Death before treatment	7 (2%)	3 (2%)	2 (1%)	2 (1%)	5 (2%)	2 (1%)
Out-patient investigation	290 (65%)	93 (63%)	98 (67%)	99 (63%)	163 (62%)	127 (68%)
PET CT	146 (33%)	15 (10%)	52 (36%)	79 (51%)	79 (30%)	67 (36%)

^a^Non-complex, ≤1 tissue diagnostic procedure and no medical delays of >3 days

^b^Complex, >1 tissue diagnostic procedures and/or medical delay of >3 days

^c^Curative radiotherapy includes concurrent radio-chemotherapy and radiotherapy alone

### Complexity

Forty-nine (11%) of patients underwent no tissue diagnostic procedure, 258 (57%) had one, 100 (22%) had two, and 42 (9%) had more than two procedures. Five hundred and ninety-five procedures were performed (279 bronchoscopies, 150 EBUS-TBNA, 166 other procedures).

Sixty-seven patients (15%) had a medical delay: 11 due to synchronous cancer, 8 had acute cardiovascular disease, 11 lung or bronchial infection, 23 poor lung- or general condition, 5 fracture or trauma, and 9 other conditions. There were delays ≥1 week due to patients’ personal preferences or no show in 13 (3%). Among these, eight had >1 tissue diagnostic procedure and/or medical delay of >3 days.

Two hundred and sixty-two patients (58%) were classified as non-complex, and there was no significant variation between years (2011: 56%, 2012: 55%, 2013: 63%; *p* = 0.37). Among complex patients, 120 (64%) had >1 tissue diagnostic procedure, 45 (24%) had medical delay of >3 days, and 22 (12%) had both >1 tissue diagnostic procedure and medical delay of >3 days. The proportion of complex among patients with NSCLC/other primary lung cancers was 49% (*n* = 156); SCLC, 25% (*n* = 16); no tissue diagnosis, 24% (*n* = 15) (*p* < 0.0001). Among patients who received treatment the proportion of complex was 44% (*n* = 161); no treatment, 30% (*n* = 26) (*p* = 0.02). There was no significant difference in the proportios who were complex in patients who had a PET CT (46%, *n* = 67) compared to those who did not have a PET CT (40%, *n* = 120) (*p* = 0.21) (Table 1).

**Fig. 1:**
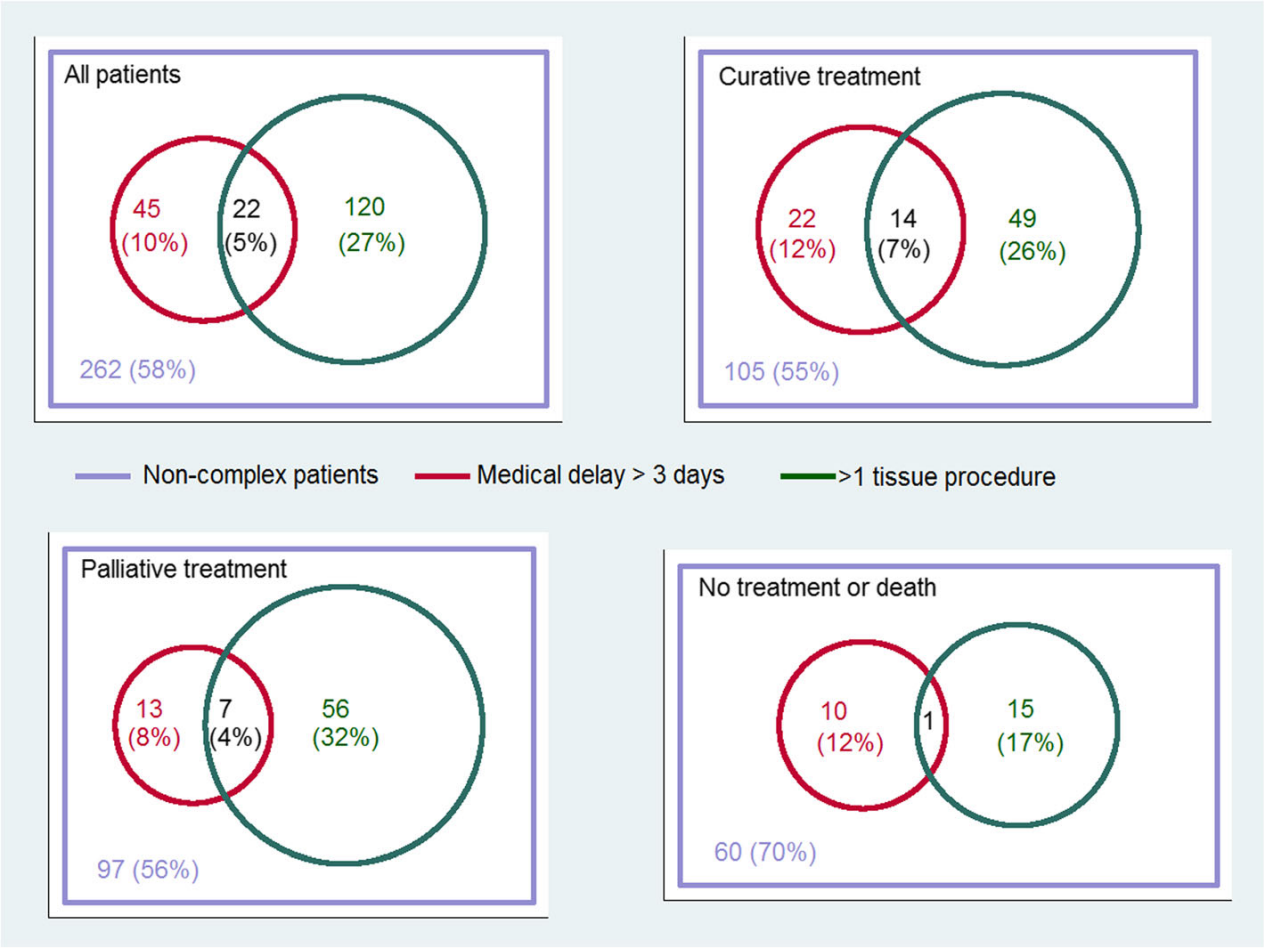
Proportions of patients with >1 tissue diagnostic procedure and medical delay >3 days. Distribution in the overall population, and split for treatment intention. Complex patients were defined as having >1 tissue diagnostic procedure and/or medical delay of >3 days.

The proportion with more than one tissue diagnostic procedure was higher among those who received treatment (33% in curative and 36% in palliative treatment), than among those who did not receive cancer treatment or died before treatment started (19%) (*p* = 0.01); while the proportions with medical delay were similar (curative treatment, 19%; palliative treatment, 12%; no treatment or death before treatment, 13% (*p* = 0.12)) (Fig. 1).

There were no significant differences in the numbers who had >1 tissue procedure depending on stage of disease (stage I: 34 (30%); stage II: 16 (38%); stage III: 38 (33%); stage IV: 54 (30%) (*p* = 0.77). The proportion of medical delays of >3 days was highest for stage II patients (stage I: 21 (19%); stage II: 12 (29%); stage III: 17 (15%); stage IV: 17 (10%) (*p* = 0.009)).

PET CT was performed in 20 (11%) of stage IV patients, and 126 (47%) of stage I-III patients (*p* < 0.0001), and the proportion was consequently higher among those receiving curative treatment (113, 59%) than those offered palliative treatment (28, 16%) or no treatment/death before treatment (5, 6%) (*p* < 0.0001).

### Intervals

Median time to treatment decision was 26 days (range: 0–283), and 247 (56%) had a decision within 28 days. Among patients who did not receive any cancer treatment, median time to that decision was 18 days (range: 0–100), and was reached in ≤28 days in 78%.

**Fig. 2:**
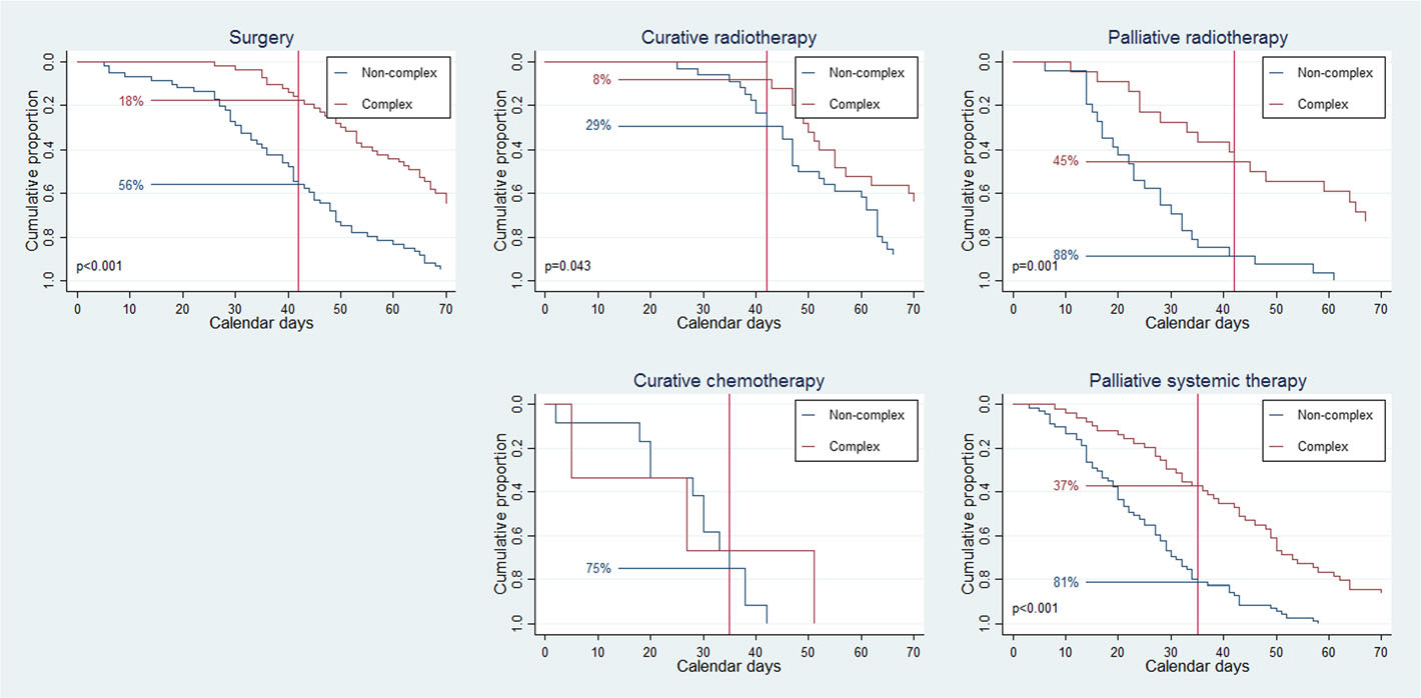
Timeliness for different treatments, split for complexity. Calendar days from the when the hospital received the referral letter for suspected lung cancer until the different treatments started. Non-complex patients were defined as having ≤1 tissue diagnostic procedure and no medical delay of >3 days, complex patients as having >1 tissue diagnostic procedure and/or medical delay of >3 days. The reference lines refer to the Norwegian recommendations for timely lung cancer treatment, which are 42 days for surgery and radiotherapy, and 35 days for systemic therapy

**Fig. 3:**
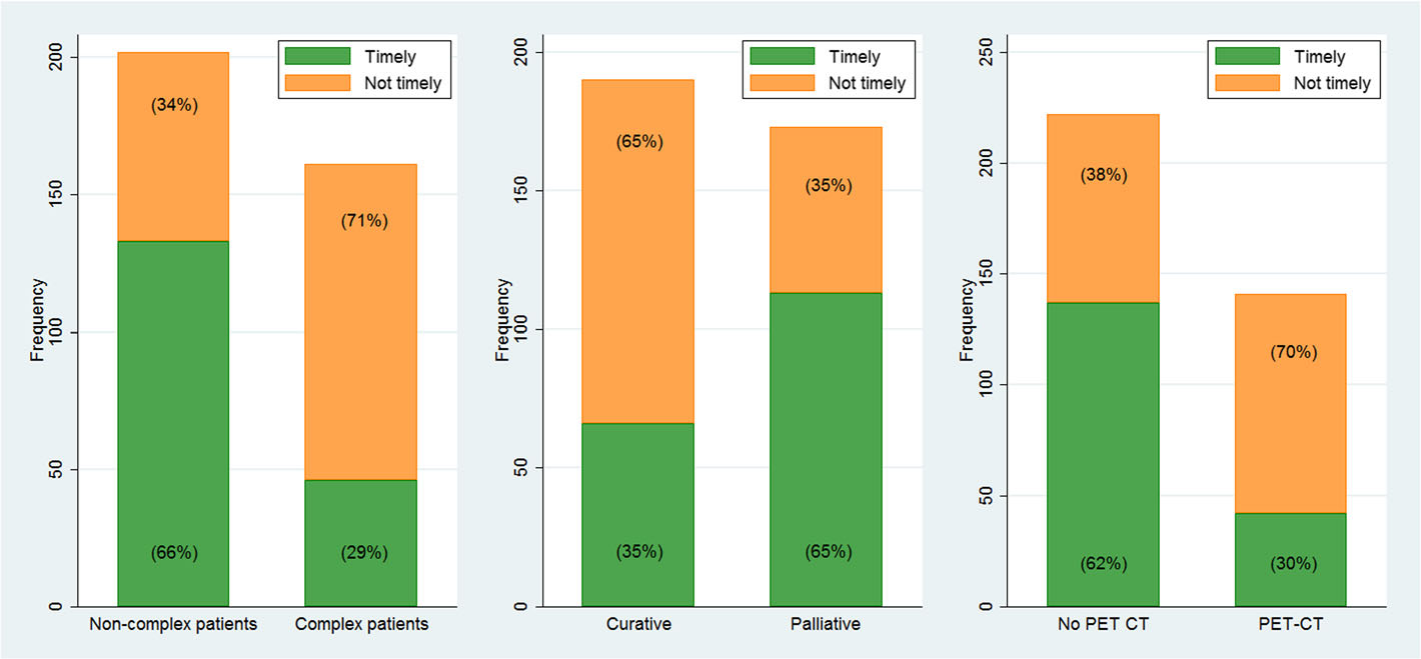
Proportions that received timely lung cancer treatment. Distributions of timely and not timely treatment in non-complex versus complex, curative versus palliative treatment, and having a PET CT versus no PET CT

In the overall population, median time to start of treatment was 42 days (range: 2–296), and 179 (49%) received timely treatment. The proportion who received timely treatment was lowest among those eligible for surgery or curative radiotherapy (Fig. 2). More patients received timely treatment among non-complex (133, 66%) than complex patients (46, 29%) (*p* < 0.0001); among those offered palliative treatment (113, 65%) than patients receiving curative treatment (66, 35%) (*p* < 0.0001); and among those who did not have a PET CT (137, 62%) than patients who underwent PET CT (42, 30%) (*p* < 0.0001) (Fig. 3).

**Fig. 4:**
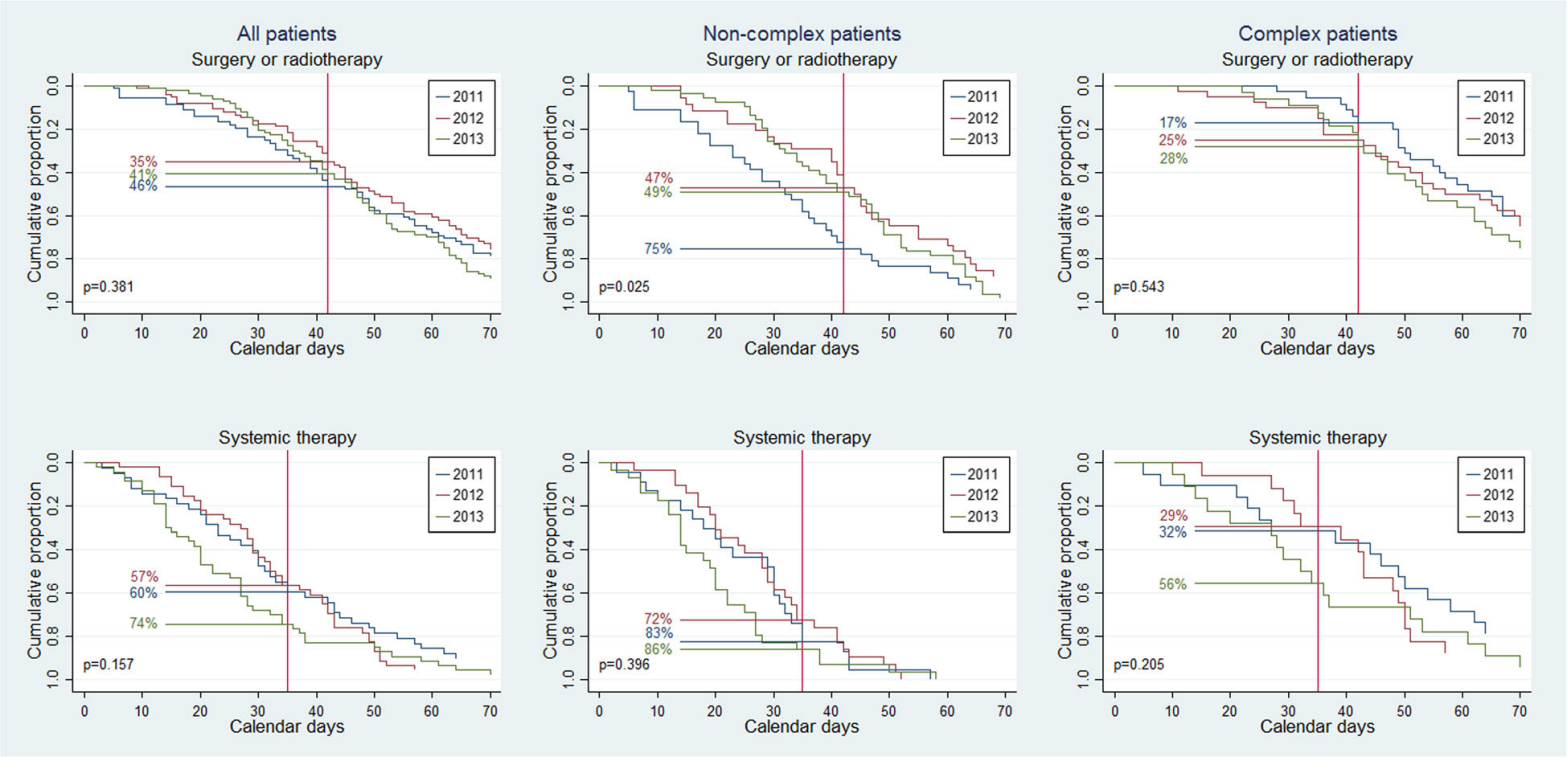
Time to treatment split for year. Calendar days from the when the hospital received the referral letter for suspected lung cancer until start of treatment. Non-complex patients were defined as having ≤1 tissue diagnostic procedure and no medical delay of >3 days, complex patients as having >1 tissue diagnostic procedure and/or medical delay of >3 days. The reference lines refer to the Norwegian recommendations for timely lung cancer treatment, which are 42 days for surgery and radiotherapy, and 35 days for systemic therapy

The proportions who received timely treatment did not vary significantly from 2011 until 2013 in the overall population, but the proportion of non-complex patients that started surgery or radiotherapy within 42 days decreased from 2011 (*n* = 27, 75%), until 2012 (*n* = 16, 47%), and 2013 (*n* = 25, 49%) (*p* = 0.03) (Fig. 4).

Median number of days until surgery or radiotherapy was 48 days (range: 5–296) among all patients (non-complex: 41 days (range: 5–145), complex: 59 days (range: 11–296)). Surgery or radiotherapy started within 42 days in 93 (41%) of all patients (non-complex: 68 (56%), complex: 25 (23%)).

Overall, 43 (37%) underwent surgery within 42 days (non-complex: 33 (56%), complex: 10 (18%); *p* < 0-0001). Corresponding numbers for curative radiotherapy was 12 (20%) (non-complex: 10 (29%), complex 2 (8%); *p* = 0.04); and for palliative radiotherapy 33 (69%) (non-complex: 23 (88%), complex: 10 (45%); *p* = 0.001) (Fig. 2).

Median number of days until systemic therapy was 29 days (range: 2–201) among all patients (non-complex: 25 days (range: 2–58), complex: 43 days (range: 5–201)). Systemic treatment started within 35 days in 86 (64%) of all patients (non-complex: 65 (80%), complex: 21 (39%)). Among those 15 patients with limited disease small-cell lung cancer who received curative chemo-radiotherapy, 12 (80%) were non-complex, and 11 (73%) received timely systemic treatment (thoracic radiotherapy was administered concurrrent with the second chemotherapy-course). Palliative systemic therapy was administered timely in 75 (63%) (non-complex: 56 (81%), complex: 19 (37%); *p* < 0.0001) (Fig. 2).

### Associations of complexity with timely treatment

Multivariate logistic regression analysis adjusted for patient characteristics (age <70/≥70, sex, outpatient/hospitalized), year and tumor characteristics (tissue diagnosis, stage) showed that complex patients were in significant risk of not timely treatment (OR, 0.16; 95% CI, 0.09-0.27). The increased risk remained significant when we adjusted for treatment intention, and whether the patient underwent PET CT or not (OR, 0.15; 95% CI, 0.09–0.26). There was no significant difference in the risk of not timely treatment between patients receiving palliative (reference category) and curative treatment (OR, 0.80; 95% CI, 0.37-1.68); while undergoing PET CT was a significant risk factor (OR, 0.32; 95% CI, 0.17-0.61). There was also no significant difference between patients with NSCLC/other primary lung cancers (reference category), SCLC (OR, 1.05; 95% CI, 0.49-2.28), and no tissue diagnosis (OR, 0.35; 95% CI, 0.12-1.04). The risk of not timely treatment was lower in stage IV than stage I-III patients (OR, 2.72; 95% CI, 1.22-6.06); and lower in hospitalized patiens than outpatients (OR, 2.35; 95% CI, 1.28-4.31).

## Discussion

In this cohort of 449 patients diagnosed with lung cancer at a regional cancer center, we found that time to start of treatment exceeded the Norwegian recommendations in 51% of those 363 that started treatment. Overall, timely treatment started in 41% of those who underwent surgery or received radiotherapy, whereas systemic therapy started within the recommended timeframe in 64%. Among the least complex patients, the timeframes where met for those who were offered systemic therapy, but not for those who underwent surgery or radiotherapy.

Interestingly, the proportion who was offered timely sugery or radiotherapy decreased from 2011 until 2013. The reason appears to be significant increase in the use of PET CT for staging of these patients due to changes in guidelines and increasing capacity in Norway. The average time for PET CT was 20 days, and in the multivariate analysis, PET CT was significantly associated with longer timeframes than recommended.

Time to treatment is a commonly used indicator of health care efficiency, but the timeframes vary in different studies and guidelines [16–18, 29]. Most commonly used are the intervals from “day of first abnormal chest image” [9, 30, 31], or “day a referral letter for suspected lung cancer was received” [6] until admission for surgery, the date of surgery, the first fraction of radiotherapy or first day of systemic therapy.

The Danish guidelines recommend that time from receiving a referral letter for suspected lung cancer until start of treatment should be ≤42 days in ≥85% of cases. In a publication from 2013 [32], they reported that the proportions of patients that started treatment within this timeframe were 63.2% (*n* = 714) for surgery, 73.5% (*n* = 687) for radiotherapy and 78.4% (*n* = 1660) for chemotherapy. The key indicator defined by the Swedish Lung Cancer Study Group is the interval from a referral letter for suspected lung cancer is received until a treatment decision is made. The goal is that a decision is made within 28 days in ≥80% of patients. In 2012–14, the goal was met in 47% (*N* = 10,369) [33], while a treatment decision was made within 28 days for 56% of our patients. The National Health Services (NHS) England recommends that patients start treatment within 62 days following an urgent general practitioner (GP) referral in ≥85% of patients. In 2013–14, 78.5% (*N* = 12,075) started treatmentwithin this timeframe [34], while 75% started treatment within 60 days in our cohort. We have not found any documentation of the rationale for the definition of the Norwegian timeframes, though they appear to be quite similar to the Danish – which are based on observations [17].

The results are not necessarily comparable due to varying lung cancer incidence [35], and there are probably differences in the organization of the health care services and availability of PET CT. Still, it appears that the situation at our hospital is similar to what was observed in Sweden and England, whereas time to surgery and radiotherapy is longer at our center than in Denmark.

The mean number of tissue diagnostic procedures was higher in Denmark [36] (1.66 vs. 1.33 in our cohort) - which might explain the higher proportion of patients with confirmed tissue diagnosis (94% vs. 86% in our cohort). The use of PET CT was much lower in our cohort (33%) than in Denmark (62%) [36]. The proportion of patients who received lung cancer treatment (81%) was higher than in Denmark (74%) [36] and England and Wales (60%) [37]. We cannot offer any obvious explanation since the study was not designed to investigate this aspect. Possible reasons include that lung cancer patients are treated in our public health care system that provides equal care for all inhabitants, and that a large proportion of patients in our area live close to the hospital.

No national guidelines recommend that all patients start treatment within the specified timeframe. Thus, it appears to be accepted that the diagnostic workup takes more time in some cases. We are, however, not aware of any studies aiming at quantifying the number of patients that should start treatment within the given timeframes. Some studies have shown that treatment is delayed if a patient has comorbidity [28, 38], or an adequate tissue sample is not obtained at first attempt [24, 25], —supporting our definition of “complex patients”. Our definition is further supported by the large difference in proportions who started timely treatment between non-complex and complex patients. We consider our defintion to be conservative. In our opinion, the health care services are not optimally organized if it takes more than 35 or 42 days to start treatment if only one tissue diagnostic procedure is required to complete diagnostic workup and there are no delays for medical reasons. One might argue that it should be possible to conduct at least two tissue procedures within the recommended timeframes. In our cohort, the results do not change much if such a cut-off value is applied – the proportion of non-complex patients receiving timely treatment changes from 66 to 58%. We have, however, chosen to use the cut-off of one tissue procedure since we presume that it is difficult to argue that this represents a non-complex patient.

The proportion of non-complex was higher among those who did not receive any lung cancer treatment. This has to be interpreted with caution. Many of these patients did not undergo an appropriate diagnostic workup since many were considered ineligible for treatment due to poor performance status or significant comorbidity, or, in some cases, because the patients did not want a complete workup.

The main limitation of our study is the retrospective design, which prohibited a uniform and systematic assessment of medical delays and delays caused by the patients’ preferences or no show. Furthermore, this is a single institution study, and not population-based. On the other hand, we are not aware of any other studies of timeliness in diagnostic workup and start of treatment for lung cancer that have assessed diagnostic complexity and medical delays. Our data are based on studies of individual medical records and not registry-based. The population represents consecutive patients diagnosed and treated at a single institution, and the patient characteristics are similar as in other unselected lung cancer polpulations [39].

Overall, the time to treatment was much longer than recommended in our cohort, − even among non-complex patients. Possible explanations include suboptimal organization, failure to comply with guidelines for diagnostic workup, low capacity for key procedures and a general lack of resources. It goes beyond the scope of this first sub-study of our project to perform value stream analyses, but we have collected these data which will be analyzed to better understand how delays can be avoided. The results will be presented in a separate article. Thus, we have currently not evaluated whether the recommended timeframes are feasible or realistic in this first sub-study of our project, but the results might provide valuable information about the proportion of patients who should receive timely lung cancer treatment. Considering that 56% of patients who started treatment were non-complex, using a conservative definition of complexity, the goal of timely treatment in 70% of cases does not appear to be unrealistic.

## Conclusion

49% of all lung cancer patients diagnosed at a university hospital started treatment within the official Norwegian timeframes. Among the least complex lung cancer patients, only 66% of patients received timely treatment. The reasons for delays needs to be identified and organization needs to be improved in order to meet the recommended timeframes.

## Additional files

Additional file 1: Medical complexity and time to lung cancer treatment. (DTA 38 kb)
Additional file 2: Medical complexity and time to lung cancer treatment. (DO 25 kb)
